# Editorial: Advanced functional materials, structures, and devices for advancing human healthcare applications

**DOI:** 10.3389/fchem.2026.1841478

**Published:** 2026-05-12

**Authors:** Feiran Li, Tianlong Li, Zhuang Hao, Dalei Jing

**Affiliations:** 1 School of Mechatronics Engineering, Harbin Institute of Technology, Harbin, China; 2 School of Mechanical Engineering and Automation, Beihang University, Beijing, China; 3 School of Engineering and Materials Science, Queen Mary University of London, London, United Kingdom

**Keywords:** advanced therapeutics, biosensing, disease diagnosis/surveillance, functional composite/nanostructures, thermoregulation

Healthcare poses a significant and enduring challenge for humanity. Recent decades have seen a substantial increase in the use of advanced, intricately designed functional materials and sensing systems, developed using cutting-edge chemical and physical principles, to address major disease challenges and enhance quality of life (Ma et al., 2021; Wang et al., 2023; Lee and Lo, 2024; Ye et al., 2024). These advancements hold great promise in areas such as smart drug delivery, disease detection and monitoring, and minimally invasive treatments (Maduraiveeran et al., 2018; Liu et al., 2022; Hao et al., 2024; Li et al., 2023; Huang et al., 2024). The resulting devices and products have immense potential to improve treatment effectiveness and reduce healthcare costs. The Research Topic “*Advanced Functional Materials, Structures, and Devices for Advancing Human Healthcare Applications*” highlights recent scientific and technological progress in this interdisciplinary field. The nine articles highlight functional materials, structures, and devices across scales that advance precision medicine, health monitoring, disease diagnosis, and therapies. These collected works can be divided into three interconnected sub-research themes: intelligent delivery and therapy, high-sensitivity diagnosis and sensing, and tissue engineering and regenerative medicine.

Micro/nanorobots (MNRs), as emerging micro- and nanocarriers, hold broad potential in drug delivery and precision medicine, making them a research hot spot. Wang et al. reviewed the potential of MNRs for treating eye diseases, focusing on their ability to bypass ocular barriers such as the blood–retinal barrier for precise drug delivery. They covered the main fabrication techniques, emphasized magnetic propulsion for reaching deep tissues, and explored applications such as tumor targeting and corneal repair.

Sui et al. showed that MNRs can target gastrointestinal therapy by overcoming barriers such as gastric acid and mucosal layers, thereby increasing oral drug delivery. They covered materials, propulsion methods (chemical, magnetic, and biohybrid), and uses in treating *H. pylori* and colorectal cancer. They also noted challenges in clinical translation, such as environmental barriers, biocompatibility, and imaging.

Fu et al. reviewed the progress of MNRs used in cancer theranostics, explaining their propulsion methods: chemical, external field, biological, and hybrid, and their roles in early detection, precise surgery, and targeted drug delivery. They highlighted the main challenges that must be addressed to unlock their full potential in cancer treatment.

Song et al. reviewed hydrogel microrobots for biomedical uses, categorizing them by source, preparation, and stimulus response. The review discussed applications such as drug delivery, tissue engineering, and minimally invasive surgery. It also highlighted challenges and future steps for clinical use and broader impact.

Tao et al. studied magnetically driven hydrogel microrobots loaded with Ro-3306 and doxorubicin, created via droplet microfluidics. These microrobots showed precise magnetic control and drug release. *In vitro* tests on MYC-dependent osteosarcoma cells showed that delivering Ro-3306 with microrobots increased the cells’ sensitivity to doxorubicin, supporting a targeted synthetic lethality approach for osteosarcoma treatment.

Early and accurate diagnosis has always been a crucial focus in healthcare research. Pei et al. developed a highly sensitive electrochemiluminescence (ECL) immunosensor for detecting the melanoma biomarker S100B. The immunosensor uses a composite electrode where platinum nanoparticles are deposited on an oxygen vacancy-rich layered double hydroxide (Pt@LDH-Ov/ITO). The electronic metal-support interaction between platinum and the oxygen vacancy-rich layered double hydroxide support significantly enhances catalytic activity in the luminol-dissolved oxygen ECL reaction, leading to strong signal amplification. The immunosensor demonstrates a wide linear range and a low detection limit for S100B, with high selectivity and stability and is applicable to spiked-serum analysis, highlighting its potential for early melanoma diagnosis.

In addition to delivery and sensing functions, advanced materials play a critical role in repairing and regenerating damaged tissues. Han et al. employed a two-step crosslinking method to develop a strong antioxidant GelMA/SA-TA composite hydrogel. Incorporating sodium acrylate and tannic acid increased its mechanical strength and capacity to neutralize free radicals. *In vitro* experiments demonstrated that the hydrogel reduced oxidative stress in rat Schwann cells by decreasing reactive oxygen species and increasing brain-derived neurotrophic factor secretion, showing its potential for peripheral nerve repair.

Sun et al. explored the use of graphene-reinforced chitosan composite tubes (G/CST) seeded with dental pulp stem cells (DPSCs) to repair facial nerve defects. The G/CST scaffold showed better mechanical strength and a more controlled degradation rate than plain chitosan tubes. In a rabbit model with a 10-mm facial nerve injury, implanting the DPSC-loaded G/CST conduit facilitated functional recovery and tissue regeneration, with results comparable to autograft transplantation.

Kuriachan and Parthiban used chemical graph theory and computational methods to calculate new domination degree-based topological indices (DTIs) for 17 heart disease drugs. They developed quantitative structure–property relationship (QSPR) models, particularly strong cubic regression models, to predict key physicochemical and absorption, distribution, metabolism, excretion, and toxicity (ADMET) properties. This work emphasizes the potential of these graph-theoretical descriptors as predictive tools in drug design.

The articles within this Research Topic highlight a diverse and vibrant research landscape. The corresponding research ranges from designing micro- and nanorobots for targeted medical procedures to developing innovative nanocomposites for highly sensitive disease detection and creating bioactive scaffolds for tissue regeneration. The collaboration of chemistry, materials science, physics, and biology is clearly evident, driving significant advances in healthcare solutions. Despite these achievements, challenges remain in areas such as biocompatibility, propulsion efficiency within living organisms, large-scale manufacturing, and the clinical application of these advanced systems. Future research is expected to focus on developing more sophisticated and multifunctional materials, integrating sensing and therapeutic functions into “theranostic” platforms, and testing these technologies in complex biological and medical settings. We hope these studies inspire continued interdisciplinary collaboration to accelerate the development and application of innovative materials and devices that improve human health.

We sincerely thank all the authors for their valuable contributions, the reviewers for their dedicated work in maintaining the scientific quality of the articles, and the editorial team at *Frontiers in Chemistry* for their support in making this Research Topic possible.
